# Can Lucifer Yellow Indicate Correct Permeability of Biological Cell Membrane under An Electric and Magnetic Field? 

**DOI:** 10.22074/cellj.2015.501

**Published:** 2015-01-13

**Authors:** Tahereh Pourmirjafari Firoozabadi, Zeinab Shankayi, Azam Izadi, Seyed Mohammad Pourmirjafari Firoozabadi

**Affiliations:** 1 Department of Physics, Varamin-Pishva Branch, Islamic Azad University, Varamin, Iran; 2 Department of Medical Physics, Faculty of Medical Science, Tarbiat Modares University, Tehran, Iran; 3 Department of Physics, Faculty of Science, K. N. Toosi University of Technology, Tehran, Iran

**Keywords:** Lucifer Yellow, Electric Field, Magnetic Field, Fluorescence Spectrum

## Abstract

The effect of external magnetic and electric fields, in the range of electroporation and magnetoporation, on Lucifer Yellow (LY) fluorescence in the absence of cells is studied. Electric-field-induced quenching and magnetic field-induced increase are observed for fluorescence intensity of LY. Regard to the fact that the variation of field-induced fluorescence, even in the absence of cells, can be observed, the application of LY, as a marker, is debatable in electroporation and magnetoporation techniques.

There has been a dramatic growth in the use of fluorescence technology by scientists from many disciplines. Although both fluorescence spectroscopy and time-resolved fluorescence were mainly considered as research tools in biochemistry and biophysics, the use of fluorescence has extended as a dominant method used substantially in biotechnology, medical diagnostics, DNA sequencing, genetic analysis, cellular and molecular imaging, and flow cytometry, as well as an indicator for cell membrane permeability in electroporation and magnetoporation (1-5).

Fluorescent markers are well-known as an analyser for cell membrane permeability in electroporation and magnetoporation, which are two practical methods for permeabilizing cell membrane (5-10). Lucifer yellow (LY) is a fluorescent molecule that does not interact with cell components; therefore, this marker has been developed as a quantitative detector of the cell membrane permeabilization (11). The quantity of LY taken up by the cells increases after the exposure of the cells to electric and magnetic fields. This change in the fluorescence spectrum is remarked as a criterion of cell membrane permeability (5-11).

Some studies have been examined the effect of external electric and magnetic fields on fluorescence to understand the mechanism of biological system reaction leading to the induced electric field by protein membranes (12-14). On the other hand, the mechanism of high electric field strength of order 100 KV/cm and synergy effects of electric and magnetic fields on fluorescence has been studied (14, 15). Nevertheless, the direct effect of magnetic and low voltage electric fields on LY has never been considered.

In the present study, LY (Sigma-Aldrich Life Science, USA) diluted in phosphate-buffered saline (PBS) with 500 μM concentration was used. The fluorescence emission was measured offline in arbitrary units on a spectrofluorometer (Shimadzu RF-5000, Japan) 40 minutes after the exposure of the LY to electric and magnetic pulses. The excitation and emission wavelengths were set at 418 and 525 nm, respectively. The number, frequency and duration of pulses in all experiments were the same and the investigated parameters were the magnetic and electric field strength. A magstim generator (Magstim Rapid, Magstim Company, Spring Gardens, Whitland, UK) was used as a magnetic stimulator and a 70 mm figure-of-eight coil with 20, 40, 60 80 and 100 % energy transfer was chosen. Electric pulses were applied to the cells by an ECT-SBDC (designed and made in the Small Business Development Center and Electromagnetic Laboratory of the Medical Physics Department of Tarbiat Modares University, Tehran, Iran). The diluted LY was placed between two parallel plate gold electrodes and exposed to 4000 electric pulses, 100 μs duration with 10, 30, 50, 70 and 90 V/cm electric field strength. All results are given as average of repeating a procedure for more than three times. Statistical analyses were performed by means of the statistical package for the social sciences (SPSS, USA) version 16. All data were tested for normality. One-way analysis of variance (ANOVA) followed by least significant difference (LSD) was performed; after that, statistical differences analysis was accomplished by t test. The p values of <0.05 were considered significant for rejection of the null hypothesis.

Fluorescence intensity of LY versus different electric and magnetic field intensities is shown in figure 1 and figure 2, respectively. The results showed a fluorescence quenching in the exposure of the electric field. On the other hand, an increase in the fluorescence intensity in the presence of magnetic field was demonstrated (2.2 T, 56 pulses with 1 Hz).

This emission intensity is a function of some parameters, such as: the amplitude of magnetic and electric fields, the number of pulses and the applied frequencies (data not shown in this paper).

**Fig 1:**
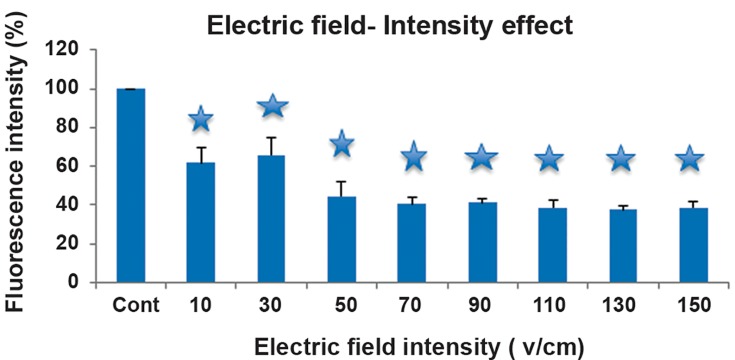
Fluorescence intensity of Lucifer Yellow (LY) versus different electric field intensity. Vertical bars represent standard deviation of the mean (*; P<0.05).

**Fig 2:**
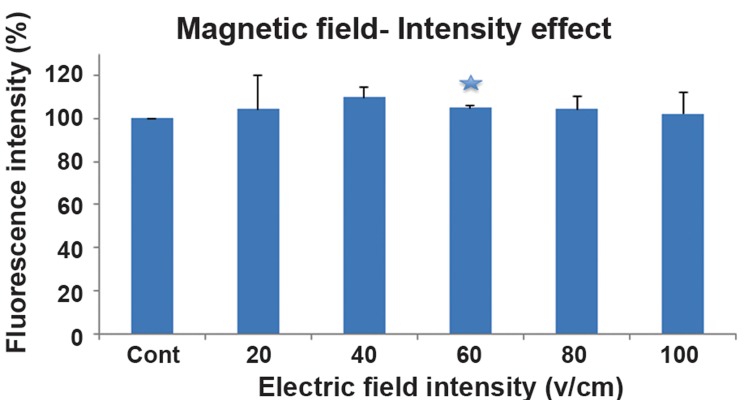
Fluorescence intensity of Lucifer Yellow (LY) versus different magnetic field power. Vertical bars represent standard deviation of the mean (*; P<0.05).

According to our findings, the changes in the fluorescence spectrum of the marker were observed even in the absence of cells; therefore, the direct effect of external electric and magnetic fields on Lucifer fluorescence was considered in this study.

The obtained results from the initial experiment, based on the fluorescent marker concentration in the presence of the low intensity electric field (less than100 V/cm) with 4000 Hz frequency (7, 8), revealed the quenching effects and its nonlinear relation with intensity. In contrast, fluorescence exposed to a magnetic field (5, 6) showed a time and frequency windowing effect.

The mechanism of the simultaneous application of electric (0.8 MVcm^-1^) and magnetic (~70G) fields on fluorescence with various methods has been studied (13, 14). In our offline study-state experiments, an increase in fluorescence intensity, under the directly induced magnetic fields (2-4 T), and fluorescence quenching, under the directly induced electric fields (10-150 Vcm^-1^), were observed. The study of the mechanism of the outcomes requires time-resolved experiments, while the purpose of current experiment is to examine the effect of magnetic and low voltage electric field-induced on LY.

Our results showed that magnetic and low voltage electric fields are two functional procedures in fluorescence spectrum.

Owing to the fact that the effects of external electric and magnetic fields on LY, in the absence of cell, are remarkable, the use of LY as a marker in electric and magnetic permeabilization is questionable. In the future papers, we will present the complementary results and discussion.
